# Preliminary Validation of a Questionnaire Covering Risk Factors for Impaired Driving Skills in Elderly Patients

**DOI:** 10.3390/geriatrics1010005

**Published:** 2016-01-08

**Authors:** Philipp Schulz, Stefan Spannhorst, Thomas Beblo, Christine Thomas, Stefan Kreisel, Martin Driessen, Max Toepper

**Affiliations:** 1 Evangelisches Krankenhaus Bielefeld, Department of Psychiatry and Psychotherapy Bethel, Research Division, Remterweg 69-71, D-33617 Bielefeld, Germany; thomas.beblo@evkb.de (T.B.); martin.driessen@evkb.de (M.D.); max.toepper@evkb.de (M.T.); 2 Evangelisches Krankenhaus Bielefeld, Department of Psychiatry and Psychotherapy Bethel, Division of Geriatric Psychiatry, Bethesdaweg 12, D-33617 Bielefeld, Germany; stefan.spannhorst@evkb.de (S.S.); stefan.kreisel@evkb.de (S.K.); 3 Clinical Centre Stuttgart, Clinic for Psychiatry and Psychotherapy for the Elderly, Prießnitzweg 24, D-70374 Stuttgart, Germany; mail@christinethomas.de

**Keywords:** Alzheimer’s disease, dementia, aging, driving fitness, risk factors

## Abstract

Due to rather unspecific statutory regulations in Germany, particularly for patients with neurodegenerative disorders, many seniors still drive despite severe driving-related cognitive deficits. An accurate assessment of driving fitness requires immense financial, personnel and temporal resources which go beyond daily clinical routines. In cooperation with a working group from Switzerland, we therefore developed the questionnaire *Safety Advice For Elderly drivers* (SAFE), an economic instrument covering different risk factors for driving safety. The main aim of the current work was a first validation of the SAFE. Twenty-two driving seniors performed the Corporal A, a test battery permitted by law to assess driving-related cognitive functions. Based upon the Corporal results and the percentile rank 16 criterion, participants were divided into cognitively impaired and unimpaired drivers. Moreover, participants were assessed using the SAFE and an extensive neuropsychological test battery. The results revealed high sensitivity and specifity scores for the SAFE suggesting that the SAFE may be a valuable and economical instrument to quantify and document individual risk factors for driving safety and to differentiate between impaired and unimpaired drivers. Notably, the results must be replicated in future studies including a larger sample, different clinical subgroups, and a practical driving lesson.

## 1. Introduction

### 1.1. Background

Aging goes along with functional and structural cerebral changes associated with decreasing cognitive performance [1]. This decrease can include various cognitive sub functions. Some of these sub functions are closely related to driving skills such as attention, working memory and cognitive flexibility [2]. As a result, driving skills also become increasingly impaired with advancing age [3]—at least cross-sectionally—which is reflected in the number of accidents in Germany: In fact, seniors are involved in fewer accidents than novice drivers, but both groups show comparable numbers of self-induced accidents involving personal injury [4]. The same applies to the number of accidents per driven kilometer where seniors and novice drivers do not only show equivalent but also higher rates than other age groups [5]. However, the reasons for increased accident rates seem to differ between novice and senior drivers: Whereas novice drivers cause more accidents as a result of higher risk tolerance or a lack of experience, the reason for increased rates of seniors may be rather found in decreased cognitive performance [4].

Particularly, the described difficulties apply to seniors suffering from neurodegenerative disorders. The most common cause of neurodegeneration is Alzheimer’s disease (AD). AD patients show specific cognitive deficits in early stages of the disease which exceed the cognitive constraints of a normal aging process. Besides memory deficits, impaired executive processes are reported [6,7,8], e.g., inhibitory dysfunctions [9]. Moreover, AD is often characterized by disorientation [10,11] and impaired symbol comprehension [12], the latter of which also affects the correct interpretation of traffic signs [13].

### 1.2. Legislation

All of the described deficits can strongly impede driving skills. Nevertheless, the regulations for AD and for dementia in general are not very specific in the German legislation (contrary to other diseases such as stroke or epilepsy). In fact, unlike as in other European countries, where the driving abilities of seniors over a certain age are regularly controlled in defined time lags, German seniors are self-responsible for their driving fitness and have to take precautions if necessary [14]. However, AD is often associated with a reduced insight into the illness (anosognosia), so that a valid judgment of their own cognitive abilities is not possible [15]. As a consequence, demented people might often still drive despite driving-related cognitive deficits. Certainly, these drivers would pose a high risk for themselves and the driving public. In the context of the demographic change and the increasing number of people suffering from dementia in the next decades, this risk weighs even more heavily [16]. Yet, there are no specific regulations for this patient group which is surprising because dementia necessarily leads to a loss of driving fitness. Another problem is that this topic is often avoided by patients and physicians in order not to burden the therapeutic relationship. Nevertheless, it is the prime duty of the physician to inform his patients about possible risk factors for driving safety and potential impairments of their fitness to drive. Only in critical cases including a serious risk to public safety, the physician is released from his duty of confidentiality and permitted to report his concerns to the authorities. In daily clinical routine, however, this option is not often exercised due to possible legal consequences.

### 1.3. How to Measure Driving Fitness?

There are different methods to gather information about a patient’s driving skills. Firstly, driving skills can be assessed by neuropsychological test batteries that are permitted by German law (*i.e.*, Testsystem Corporal, Wiener Testsystem, Action-Reaction-Testsystem) to examine driving-related cognitive functions. In this context, German standards and statutory regulations propose the percentile rank 16 criterion for all individuals with a valid driving license and independent of age [17]. This criterion claims that only individuals who reach a percentile rank of at least 16 in all subtests of the respective test battery are regarded as fit to drive. However, possible deficits (percentile rank < 16) might be situational and could be compensated for in everyday life. A more realistic method to assess driving fitness therefore is a practical driving lesson. In this driving lesson, a driving instructor and a traffic psychologist judge the individual’s fitness to drive in a real setting. However, such judgments are at least partly subjective. Moreover, driving lessons do not cover unusual events that require an exceptionally fast reaction from the driver (e.g., child on the street). Taken together, the described methods have assets and drawbacks. Consequently, the combination of an interview with a traffic psychologist, a traffic medical investigation, psychometric diagnostics and a practical driving lesson is regarded as the gold standard in Germany. However, such procedures require immense financial, personnel and temporal resources which go beyond daily clinical routines. This notwithstanding, a driver can voluntarily have his driving proficiency evaluated by official institutions but must carry the financial burden. Consequently, this option is not often taken.

### 1.4. Rationale of the Study

For all of the reasons mentioned above, our working group has developed the questionnaire *Safety Advice For Elderly drivers* (SAFE; please see supplementary material) with the main aim to improve the quality of consultation for seniors in our memory clinic [18]. The SAFE is based on a comparable project in Switzerland [19] and was adapted by our working group considering literature and clinical experience. The SAFE covers possible risk factors for driving safety including an accident history, constraints in daily functioning as well as visual, motor, cognitive and clinical risk factors (Section 2.4). The objective of the current pilot study was a first validation of the SAFE. In this context, it was of particular relevance whether and how accurate the SAFE would be able to differentiate between senior drivers with impaired and unimpaired driving-related cognitive functions. In addition, diagnostic accuracy of common neuropsychological tests in differentiating between impaired and unimpaired drivers (based upon Corporal A results and percentile rank 16 criterion) should be examined.

## 2. Methods

### 2.1. Participants

The study included 22 driving seniors (Table 1) who initially consulted our memory clinic because of subjective cognitive deficits. Before driving assessment, subjects were informed about the specific experimental procedures and provided a written declaration of consent. All procedures were in accordance with the Declaration of Helsinki. Post-hoc diagnoses were given a few weeks after study participation.

**Table 1:** Sample Characteristics. Mean values and standard deviations (in brackets) are displayed.

*N*	22
sex (female/male)	7/15
age in years (SD)	71.6 (8.3)
minimum	56
maximum	88
school education in years (SD)	9.9 (1.9)
minimum	8
maximum	13
MMSE score (SD)	27.3 (2.6)
minimum	19
maximum	30
Post-hoc diagnoses (*n*)	
no psychiatric diagnosis	8
AD dementia	3
Vascular dementia	1
MCI	4
Affective disorder	6

*N* = number of participants; SD = standard deviation; MMSE = Mini Mental Status Examination; AD = Alzheimer’s disease; MCI = mild cognitive impairment.

### 2.2. Corporal A

The participants’ driving-related cognitive abilities were examined by a trained psychologist using the Corporal A (Vistec AG, Olching, Germany). The Corporal A is a computerized test battery that is permitted by law to examine driving-related cognitive functions [20]. It is broadly used in official assessment centers for driving fitness and not limited to certain target groups (e.g., older driver). The Corporal is employed if there is a suspicion of mental impairment that may impede driving fitness. Duration of the Corporal A is approximately 20 min. The Corporal A includes three different subtests assessing alertness, selected attention, and divided attention, respectively. Participants were provided with a keypad and had to react to visual stimuli displayed on a computer screen. Performance quality in each of these subtests was reflected by a combined score of speed and accuracy. Based on these scores and the percentile rank 16 criterion using age-independent standard values [17], participants were classified into “impaired” and “unimpaired” drivers. As mentioned in Section 1.2, these regulations claim that only participants who reach a percentile rank of at least 16 in all of the applied Corporal A subtests are regarded as unimpaired drivers [17].

### 2.3. Neuropsychological Data

Moreover, all participants underwent an extensive neuropsychological examination. The duration of this examination varied between 60 and 75 min depending on the cognitive status of the respective participant. Global cognitive functioning was assessed with the Mini Mental Status Examination (MMSE; [21]). Verbal learning, verbal memory retrieval, and verbal recognition were reflected by the test scores in the German version of the Rey auditory verbal learning test (RAVLT; [22]), whereas nonverbal memory retrieval was assessed with the subtest Constructional Praxis II of the Consortium to Establish A Registry for Alzheimer’s Disease (CERAD; [23,24]). Semantic memory was assessed using a 15-items version of the Boston naming test (BNT; [25]) and a category fluency task (animal fluency; [26]). Processing speed was measured by part A of the trail making test (TMT; [27,28]) and spatial abilities by the clock drawing test [29,30]. Code shifting was assessed with a number transcoding task of the dementia detection test (DemTect; [31]) whereas cognitive flexibility was estimated by the test scores in part B of the trail making test [27,28]. In all tests, higher scores reflected better performances, except for the number transcoding task, RAVLT false positives, the clock drawing test and the TMT in which higher scores reflected lower performances (errors and time, respectively).

### 2.4. Safety Advice For Elderly Drivers (SAFE)

Finally, one of three attending physicians discussed the findings with the patient and, in some of the cases, with a close relative. Afterwards, the physician rated the different risk factors for driving safety by using the SAFE [18]. The duration of this procedure was approximately 20 min. The SAFE includes different risk factors for driving safety. Besides an accident history and an examination of basic activities of daily living [32], the SAFE includes an identification of visual (visual acuity, field of view), motor (cervical spine) and cognitive (e.g., cognitive flexibility) constraints as well as the documentation of relevant diseases (e.g., Parkinson disease, stroke), symptoms (e.g., impulsiveness) and medication (e.g., psychotropic). Moreover, dementia etiology, disease severity and other driving-relevant risk factors (e.g., right-left disorientation) are inquired. Each of the different risk factors represents either a low, medium or high risk for driving safety and can be marked with a cross on the questionnaire (in the respective circles). The SAFE is comparable to the ADReS (Assessment of Driving Related Skills) test battery that is recommended by the American Medical Association to assess driving-related skills in older drivers with and without cognitive impairments [33].

Based on the weighted SAFE risk factors, the individual risk profile, specific interpretation guidelines (*i.e.*, one low and two medium risk factors), and the clinical impression, the attending physician rated the total risk on a scale from one to five (low-medium-high-very high-unclear). Collected scores were the total risk as rated by the physicians, the total number of identified risk factors, and a sum score based upon the weighting of the identified risk factors (low = 1, medium = 2, high = 3).

The attending physicians were blinded to the Corporal A results and the results of the neuropsychological test battery (except for MMSE and TMT-B results that were part of the SAFE).

### 2.5. Statistics

Data were analyzed using SPSS Statistics 20.0. In a first step, participants were classified into a group of impaired drivers and a group of unimpaired drivers according to the Corporal A test scores and the percentile rank 16 criterion (Section 2.2). Due to the small sample size, we used Kendalls tau-b rank correlations to analyze the relationships between the subscores of the SAFE and overall Corporal A performance as well as between neuropsychological test scores and overall Corporal A performance. Overall Corporal A performance was reflected by the mean value of the combined Corporal A subscores. To control for possible age effects, we additionally calculated partial rank correlations [34]. To examine the diagnostic accuracy of the SAFE and the single neuropsychological tests in differentiating between impaired and unimpaired drivers, receiver operating characteristic (ROC) curve analyses were run using area under the curve. These analyses identify the cut points providing the best balance between sensitivity and specificity. For the SAFE, ROC analyses were run for all collected scores (total risk, number of risk factors, sum score of weighted risk factors).

## 3. Results

### 3.1. Corporal A

**Figure 1:**
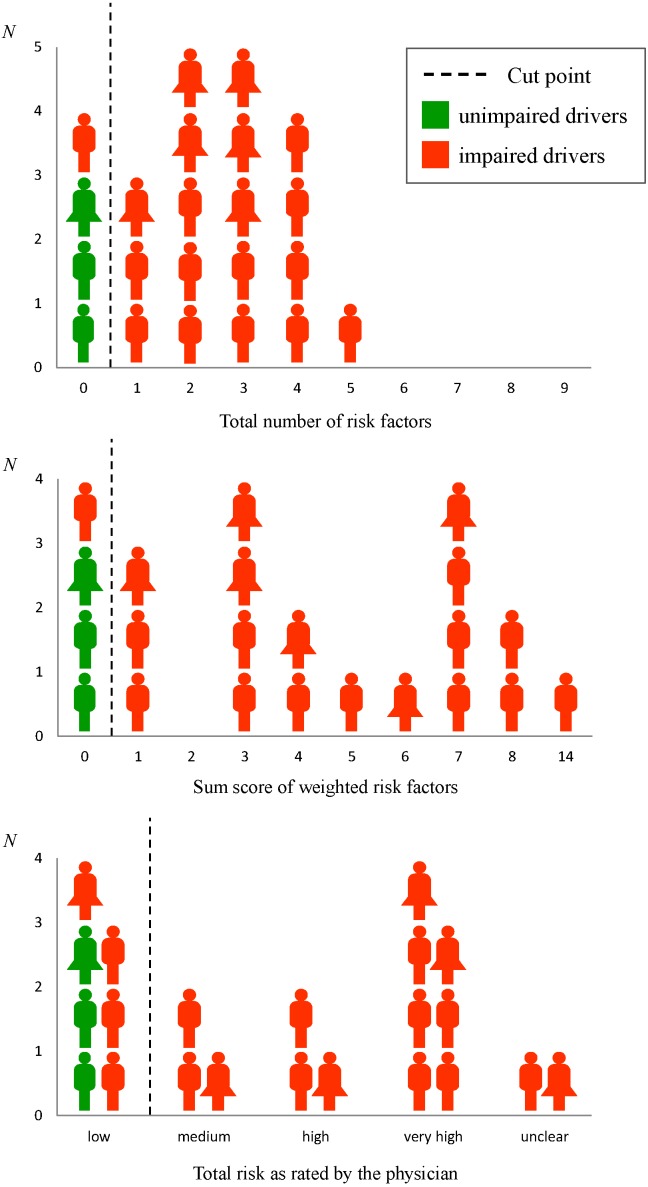
Sensitivity and specifity of the different SAFE scores (total number of risk factors, sum score of weighted risk factors, total risk as rated by the physicians), depending on the cut points providing the best balance between sensitivity and specifity. Drivers with impaired driving-related cognitive functions according to the Corporal A results and the percentile rank 16 criterion are marked in red; unimpaired drivers are marked in green. *N* = number of participants.

Results showed that 86% (*n* = 19) of the participants were identified as impaired drivers and 14% (*n* = 3) as unimpaired drivers according to the Corporal A results and the percentile rank 16 criterion (Figure 1). Descriptive data is shown in Table 2.

**Table 2:** Descriptive data of impaired and unimpaired drivers due to the Corporal A results and the percentile rank 16 criterion. Mean values and standard deviations (in brackets) are displayed.

	Unimpaired	Impaired
*N*	3	19
age (SD)	60.7 (4.7)	73.4 (7.4)
sex (female/male)	1/2	5/14
school education in years (SD)	10.7 (1.5)	9.8 (1.9)
SAFE total risk (SD)	0	1.8 (1.3)
Number of SAFE risk factors (SD)	0	2.6 (1.3)
SAFE risk factor score (SD)	0	4.8 (3.4)
MMSE score (SD)	29.3 (0.6)	26.9 (2.7)
TMT-B score (SD)	74 (34)	192.6 (121.6)
Post-hoc diagnoses		
no psychiatric diagnosis	2	6
AD dementia	0	3
Vascular dementia	0	1
MCI	0	4
Affective disorder	1	5

*N* = Number of participants; SD = standard deviation; AD = Alzheimer’s disease; MCI = mild cognitive impairment; MMSE = Mini Mental Status Examination; TMT-B = Trail Making Test part B.

### 3.2. SAFE

Correlation analyses revealed significant correlations between all of the SAFE subscores and the overall Corporal A performance (Table 3). If being controlled for age, the total risk as rated by the physician and the total number of SAFE risk factors showed marginally significant correlations with the overall Corporal A performance.

**Table 3:** Descriptive data of SAFE risk factors and correlations with Corporal A performance.

SAFE Subscores and Risk Factors	Descriptive Data			Correlations with Overall Corporal A Performance ^1^	
	*n*	M	SD	Zero Order	Controlled for Age
SAFE subscores					
Total risk ^2^	22	1.5	1.3	−0.55 **	−0.41 ^+^
Number of risk factors	22	2.2	1.5	−0.49 **	−0.39 ^+^
Risk factor score	22	4.2	3.6	−0.43 **	−0.33
Frequency of SAFE risk factors					
TMT-B	15				
MMSE	13				
Avoidance behavior	8				
Anosognosia/thought disorder *etc.*	3				
Accidents/traffic offences/police controls due to driving behavior (last 2 years)	2				
Passenger feels unsafe	2				
Daytime sleepiness	2				
Impairment BADL	1				
Limited head rotation, but >45°	1				
Psychotropic substances (uptitration phase)	1				
Mild Alzheimer’s dementia	1				

^1^ Overall Corporal A performance was reflected by the mean value of the three combined Corporal A subscores. Higher scores reflected better performance; ^2^ Total risk was rated on a 4-point-scale from 0 = low risk to 3 = very high risk; ** *p* < 0.01, ^+^ *p* < 0.10; MMSE = Mini Mental Status Examination; TMT-B = Trail Making Test part B; BADL = basic activities of daily living.

ROC curve analyses revealed high sensitivity and specifity scores for all collected SAFE scores (Table 4, Figure 1). The number of risk factors and the sum score of weighted risk factors differentiated between impaired and unimpaired drivers with a sensitivity of 95% and a specifity of 100%. For both scores, the positive predictive value was 100% with a negative predictive value of 75%. The total risk as rated by the physicians showed a sensitivity of 79%, a specifity of 100%, a positive predictive value of 100%, and a negative predictive value of 43%. Consequently, the different scores correctly identified between 15 and 18 of the impaired drivers (19) and all of the unimpaired drivers as per Equation (3).

**Table 4:** Diagnostic accuracy of different SAFE subscores in differentiating between impaired and unimpaired drivers depending on specific cut points.

SAFE Subscores	Unimpaired Drivers	Impaired Drivers	Sensitivity (%)	Specifity (%)	PPV (%)	NPV (%)
Total risk	low	≥medium	79	100	100	43
Number of risk factors	0	≥1	95	100	100	75
Risk factor score	0	≥1	95	100	100	75

PPV = positive predictive value; NPV = negative predictive value.

### 3.3. Neuropsychological Tests

The results of the neuropsychological examination as well as the correlations with the overall Corporal A performance are displayed in Table 5.

**Table 5:** Neuropsychological test results and correlations with overall Corporal A performance.

Neuropsychological Tests	M	Md	Min	Max	SD	Correlation with Corporal A ^1^
MMSE	27.3	28	19	30	2.6	0.54 **
RAVLT learning	41.4	43	12	66	14.6	0.50 **
RAVLT recall	6	7	0	15	4.8	0.43 **
RAVLT recognition	12.3	14	3	15	3.2	0.42 *
RAVLT false positives	6.2	4	0	20	6.3	−0.44 **
Constructional Praxis II	5.9	7	0	11	3.4	0.40 *
BNT	14	15	10	15	1.6	0.29
Category Fluency Task	19.3	19.5	9	33	6	0.31 *
TMT-A	45.9	44.5	18	87	16.9	−0.60 **
Clock Drawing Test	0.23	0	0	1	0.43	−0.20
Number Transcoding	0.5	0	0	4	0.96	−0.28
TMT-B	176.5	132.5	41	517	120.5	−0.50 **

^1^ Overall Corporal A performance was reflected by the mean value of the three combined Corporal A subscores. Higher scores reflected better performance; RAVLT = Rey auditory verbal learning test; RAVLT learning = total number of words learned from trial 1 to 5; RAVLT recall = total number of words correctly recalled after a delay of 30 min; RAVLT recognition = number of words correctly recognized after a delay of 30 min; RAVLT false positives = false positive responses during word recognition; Constructional Praxis II = sum score for correctly recalled geometric figures after a delay of 10 min; BNT = Boston Naming Test (total score); Category Fluency Task = total number of animals within 60 s; TMT = Trail making test (time in seconds); MMSE = Mini Mental Status Examination; ** *p* < 0.01, * *p* < 0.05.

Results for different neuropsychological test scores revealed sensitivity scores between 21% and 89% and specifity scores between 67% and 100% (Table 6). The highest accuracy scores were found for the TMT-B and the number of false positive responses during word recognition (RAVLT): The TMT-B showed a sensitivity of 79%, a specifity of 100%, a positive predictive value of 100% and a negative predictive value of 43%. The number of false positive responses during word recognition (RAVLT) showed a sensitivity of 83%, a specifity of 100%, a positive predictive value of 100% and a negative predictive value of 50%. All of the other neuropsychological tests scores showed either sensitivity or specifity scores under 70%.

**Table 6:** Diagnostic accuracy of different neuropsychological test scores subscores in differentiating between impaired and unimpaired drivers depending on specific cut points.

Neuropsychological Tests	Unimpaired Drivers	Impaired Drivers	Sensitivity (%)	Specifity (%)	PPV (%)	NPV (%)
MMSE	≥29	≤28	68	100	100	33
RAVLT learning	≥54	≤53	89	67	94	50
RAVLT recall	≥11	≤10	89	67	94	50
RAVLT recognition	≥14	≤13	56	100	100	27
RAVLT false positives	≤1	≥2	83	100	100	50
Constructional Praxis II	≥8	≤7	78	67	93	33
BNT	15	≤14	53	100	100	25
Category Fluency Task	≥24	≤23	84	67	94	40
TMT-A	≤37	≥38	68	100	100	33
Clock Drawing Test	0	≥1	21	67	80	12
Number Transcoding	0	≥1	32	67	86	13
TMT-B	≤116	≥117	79	100	100	43

MMSE = Mini Mental Status Examination; RAVLT = Rey auditory verbal learning test; RAVLT learning = total number of words learned from trial 1 to 5; RAVLT recall = total number of words correctly recalled after a delay of 30 min; RAVLT recognition = number of words correctly recognized after a delay of 30 min; RAVLT false positives = false positive responses during word recognition; Constructional Praxis II = sum score for correctly recalled geometric figures after a delay of 10 min; BNT = Boston Naming Test (total score); Category Fluency Task = total number of animals within 60 s; TMT = Trail making test (time in seconds).

## 4. Discussion

The results of the current work revealed that the SAFE seems to be a valuable and economic instrument to differentiate between cognitively impaired and unimpaired drivers with high diagnostic accuracy.

### 4.1. Corporal A and Percentile Rank 16 Criterion

The Corporal A results suggest that driving abilities are affected by age, disease and cognition: Impaired drivers were older, 13 out of 19 impaired drivers had psychiatric diagnoses and impaired drivers showed poorer cognitive performance than unimpaired drivers. Interestingly, the majority of drivers did not reach a percentile rank of at least 16 in all of the Corporal A sub tests. Consequently, consistent with German standards and statutory regulations, 19 out of 22 participants were classified as “impaired drivers” regarding their driving-related cognitive functions. At first sight, these findings imply that there are many drivers who should not drive anymore because they do not meet the cognitive requirements. However, some studies suggest that the percentile rank 16 criterion might be too rigorous to judge driving skills. In fact, many drivers who did not meet this criterion showed unimpaired driving skills in a practical driving lesson [35,36,37]. These findings propose that driving-related cognitive performances in the laboratory are sometimes situational and can be compensated in a real setting. Such compensatory mechanisms may be driving experience, anticipatory driving, a realistic self-awareness or technical features of the vehicle [38]. In Germany, a combination of an interview with a traffic psychologist, a traffic medical investigation, psychometric diagnostics and a practical driving lesson is therefore regarded as the gold standard to identify unfit drivers. Following this argumentation, some of the impaired drivers in the current study might have been fit to drive after all because they might have been able to compensate for their deficits in everyday life.

### 4.2. SAFE

However, given the Corporal A results and the percentile rank 16 criterion as decision criterions for driving fitness, the current study revealed that the SAFE was able to differentiate between cognitively impaired and unimpaired drivers with high diagnostic accuracy. In fact, the different scores showed sensitivities between 79% and 95% with a specifity of 100%. Consequently, most of the impaired drivers could be correctly identified as well as all of the unimpaired drivers. Interestingly, the total risk as rated by the physicians showed lower accuracy than the other scores, probably because the total risk estimation of the attending physicians does not only consider the number and severity of different risk factors but also an individual risk profile and possible compensatory resources. Notably, the SAFE subscores were correlated with age indicating that older participants showed a higher number of risk factors. This finding suggests that higher age may be a risk factor for driving safety per se, which is in line with previous findings [3,35]. However, two of the SAFE subscores were still correlated with Corporal A performance after being controlled for age indicating an age-*independent* relationship between the SAFE and cognitive driving fitness as well.

Our findings suggest that the SAFE may be a valuable instrument to detect impaired drivers and to quantify individual risk factors for driving safety. The quantification of risk factors offers the chance to convince cognitively impaired drivers of their constraints. In particular, this applies to demented people who are not aware of their disease and the related cognitive dysfunction. In this context, the SAFE can provide the basis for a constructive discussion between physician and patient about possible training methods or quitting driving and organizing possible transport alternatives. Finally, the SAFE facilitates the documentation of a physician’s consultation addressing the driving fitness of a patient. Such documentation can be very important to protect the physician against legal consequences, particularly if an impaired driver does not respect the judgment of the physician and causes an accident. Taken together, the SAFE seems to be a helpful and economical instrument to improve the consultation quality of driving seniors. In a greater context, it affords the opportunity to reduce the risk impaired drivers pose to themselves and to the driving public.

### 4.3. Neuropsychological Tests

Analysis of neuropsychological data showed that most of the single neuropsychological tests were not able to differentiate between impaired and unimpaired drivers with satisfying diagnostic accuracy because they either showed low sensitivity or low specifity scores. Exceptions were the TMT-B and the number of false positive responses during verbal recognition (RAVLT). The respective test scores showed sensitivity scores between 79% and 83% at a specifity of 100%. As expected, one of the highest diagnostic accuracy scores among all neuropsychological tests was found for the TMT-B. This result is in line with previous findings [39,40] suggesting that cognitive flexibility is a sensitive marker for driving ability and that the TMT-B may be the first choice if only one neuropsychological test can be conducted. Of course, this information is not sufficient to judge whether an individual is fit to drive or not, but it may point toward a possible risk. Moreover, the number of false positive responses during word recognition proved to be rather accurate indicating that memory functions are also associated with driving fitness.

Noteworthy, the Corporal A and other licensed test batteries examining driving-related cognitive sub functions comprise exclusively attention tests. However, the current results confirm that driving abilities are also associated with executive and memory functions. In particular, cognitive flexibility was found to be associated with driving fitness [41] suggesting that the ability to flexibly shift between different sources of information is necessary for driving safety, particularly in complex traffic situations. The same applies to memory functions. A prominent example may be a senior driver who suffers from early AD typically does not show attentional deficits, but has difficulties remembering the meaning of certain traffic signs. A correct interpretation of these signs by semantic-associative processes is often not possible either, because many of these signs are too abstract to be understood without mnemonic information. Moreover, symbol comprehension was found to be impaired in AD [12] which also applies to traffic symbols [13]. Together, these findings suggest that future approaches differentiating between fit and unfit drivers should not exclusively focus on impaired attentional processes but also include executive and memory tasks. In fact, the new Corporal version will comprise such sub tests.

### 4.4. Conclusions and Limitations

Overall, the results of the current work suggest that the SAFE may be a valuable and economical instrument to quantify and document individual risk factors for driving safety, and to differentiate between impaired and unimpaired drivers. It can provide the basis for constructive discussions between patient and attending physician about training methods or possible transport alternatives and may lower the risk for the patient himself and the driving public.

However, the current work has some major limitations. First of all, it must be stated that the sample size is far too small to draw general conclusions. Moreover, the sizes of the groups of impaired and unimpaired drivers greatly differed. In addition, the sample was quite heterogeneous by including healthy seniors and patients with different diagnoses like MCI, Dementia and affective disorders. Finally, the decision criterion for driving fitness was represented by the Corporal test system and the percentile rank 16 criterion and not by the gold standard including, for instance, a practical driving lesson.

For all of these reasons, the present study can only be regarded as a pilot project or pre-validation based on preliminary results. Nevertheless, our findings suggest that either the percentile rank 16 criterion is far too rigorous or that there are many impaired drivers who still drive. In future, the current results must be replicated in a larger sample including groups of healthy seniors and different clinical groups of well-diagnosed patients. Most importantly, such a follow-up validation should use the gold standard (psychometric tests, practical driving lesson, *etc.*) to classify individuals into fit or unfit drivers. Finally, the SAFE could be further optimized by including additional sensitive risk factors and by developing an algorithm for objective total risk estimation.

## Supplementary Materials

The following are available online at www.mdpi.com/2308-3417/1/1/5/S1, German and English versions of the SAFE questionnaire.
