# Supplementary material for: Preliminary Validation of a Questionnaire Covering Risk Factors for Impaired Driving Skills in Elderly Patients
Source: Geriatrics (Basel). 2016 Jan 8;1(1):5. doi: 10.3390/geriatrics1010005 (PMC6371095; doi:10.3390/geriatrics1010005)
Supplement: Supplementary file 1 [file geriatrics-01-00005-s001.zip › SAFE_english.pdf]

# – SAFE –

Safety Advice For Elderly drivers

Name: ________________________________ Date of birth: _______________________ Sex: ☐ f ☐ m

Active driver? ☐ yes ☐ no If not, why? ____

Risk factor	Risk level				
	unclear	no risk	low	medium	high
**1 Accident history**					
1.1 Accidents/traffic offences/police controls due to driving behavior (last 2 years)	○	○	☒	○	☒
1.2 Passenger feels unsafe	○	○	☒	○	☒
1.3 Avoidance behavior (e.g. driver does not drive during darkness anymore)	○	○	☒	○	☒
**2 Activities of daily living**					
2.1 Impairment BADL	○	○	☒	☒	○
**3 Vision**					
3.1 Visual acuity (eye chart < 60 %)	○	○	☒	☒	○
3.2 Field of view < 140 degrees/double vision/neglect	○	○	☒	☒	○
**4 Cervical spine mobility**					
4.1 Limited head rotation, but > 45 degrees	○	○	○	☒	☒
4.2 Head rotation < 45 degrees	○	○	☒	☒	○
**5 Diseases, symptoms, medication**					
5.1 Parkinson's disease	○	○	☒	○	☒
5.2 Epilepsy (asymptomatic < 12 months and/or syncope < 6 months)	○	○	☒	☒	○
5.3 Stroke (hemorrhage or ischemia) < 6 months	○	○	☒	☒	○
5.4 Diabetes Mellitus including hypoglycemia and/or relevant somatic impairments	○	○	☒	○	☒
5.5 Addiction (alcohol/benzodiazepine)	○	○	☒	☒	○
5.6 Psychotropic substances, sedative (long-term therapy)	○	○	☒	○	☒
5.7 Psychotropic substances (uptitration phase)	○	○	☒	☒	○
5.8 Impulsive/aggressive behavior	○	○	☒	☒	○
5.9 Daytime sleepiness (ESS)	○	○	☒	☒	○
**6 Global cognitive functioning**					
6.1 MMSE: 25-28 points	○	○	○	☒	☒
6.2 MMSE: 22-24 points	○	○	☒	○	☒
6.3 MMSE: < 22 points	○	○	☒	☒	○
**7 Cognitive flexibility**					
7.1 Trail Making Test B: 120-139 seconds	○	○	○	☒	☒
7.2 Trail Making Test B: 140-180 seconds	○	○	☒	○	☒
7.1 Trail Making Test B: > 180 seconds or cannot be completed	○	○	☒	☒	○
**8 Dementia etiology and severity**					
8.1 Mild Alzheimer's dementia (MMSE > 24 points, TMT B < 140 seconds)	○	○	○	☒	☒
8.2 Frontotemporal dementia	○	○	☒	☒	○
8.3 Dementia with Lewy bodies	○	○	☒	☒	○
8.4 Parkinson's dementia	○	○	☒	☒	○
**9 Other risk factors**					
9.1 Anosognosia/thought disorder/right-left disorientation etc.					

Total risk estimation for driving safety

low	medium[1]	high[2]	very high[3]	unclear
○	○	○	○	○

Interpretation guidelines: [1]maximal 2 low risk factors or 1 medium risk factor, [2]more than 2 low risk factors or more than 1 medium risk factor, [3]at least 1 high risk factor

EvKB, Department of Geriatric Psychiatry
