# Supplementary material for: Preliminary Validation of a Questionnaire Covering Risk Factors for Impaired Driving Skills in Elderly Patients
Source: Geriatrics (Basel). 2016 Jan 8;1(1):5. doi: 10.3390/geriatrics1010005 (PMC6371095; doi:10.3390/geriatrics1010005)
Supplement: Supplementary file 1 [file geriatrics-01-00005-s001.zip › SAFE_german.pdf]

# – SAFE –

Seniorenberatung Aufgrund Fahreignungsrelevanter Einschränkungen

Name: ________________________________ Geb.-Dat.: _______________________ Geschlecht: ☐ w ☐ m

Motorisierte Teilnahme am StV? ☐ ja ☐ nein Wenn nein, warum nicht? ________________________________

Risikofaktor	Risikolevel				
	unklar	kein Risiko	niedrig	mittel	hoch
**1 Fahranamnese**					
1.1 Unfälle/Verkehrsdelikte/Polizeikontrollen wg. auffälliger Fahrweise (letzte 2 Jahre)	○	○	X	○	X
1.2 Beifahrer fühlt sich unsicher	○	○	X	○	X
1.3 Vermeidungsstrategien (z.B. Fahrer fährt nicht mehr bei Dunkelheit etc.)	○	○	X	○	X
**2 Alltagsaktivitäten**					
2.1 Beeinträchtigung BADL	○	○	X	X	○
**3 Sehen**					
3.1 Sehschärfe (Sehtafel < 60 %)	○	○	X	X	○
3.2 Gesichtsfeld < 140 Grad/Doppelbilder/Halbseitenneglect	○	○	X	X	○
**4 HWS-Beweglichkeit**					
4.1 Kopfdrehen eingeschränkt, aber > 45 Grad	○	○	○	X	X
4.2 Kopfdrehen < 45 Grad	○	○	X	X	○
**5 Erkrankungen, Symptome und Medikamente**					
5.1 Morbus Parkinson	○	○	X	○	X
5.2 Epilepsie (asymptomatisch < 12 Monate und/oder Synkope < 6 Monate)	○	○	X	X	○
5.3 Schlaganfall (Blutung oder Ischämie) < 6 Monate	○	○	X	X	○
5.4 Diabetes Mellitus mit Hypoglykämie(n) und/oder relevanten körperl. Spätfolgen	○	○	X	○	X
5.5 Hinweise auf Suchterkrankung (Alkohol/Benzodiazepine)	○	○	X	X	○
5.6 Psychotrope Substanzen, sedierend (Dauertherapie)	○	○	X	○	X
5.7 Psychotrope Substanzen (Eindosierungsphase)	○	○	X	X	○
5.8 Deutlich impulsives/aggressives Verhalten	○	○	X	X	○
5.9 Tagesschläfrigkeit (ESS)	○	○	X	X	○
**6 Globales kognitives Leistungsniveau**					
6.1 MMST: 25-28 Punkte	○	○	○	X	X
6.2 MMST: 22-24 Punkte	○	○	X	○	X
6.3 MMST: < 22 Punkte	○	○	X	X	○
**7 Kognitive Flexibilität**					
7.1 Trail Making Test B: 120-139 Sekunden	○	○	○	X	X
7.2 Trail Making Test B: 140-180 Sekunden	○	○	X	○	X
7.1 Trail Making Test B: > 180 Sekunden oder nicht leistbar	○	○	X	X	○
**8 Demenzätiologie und Schweregrad**					
8.1 Leichte Alzheimer-Demenz (MMST > 24 Punkte, TMT B < 140 Sekunden)	○	○	○	X	X
8.2 Frontotemporale Lobärdegeneration (Verhaltensvariante)	○	○	X	X	○
8.3 Lewy-Body-Demenz	○	○	X	X	○
8.4 Parkinson-Demenz	○	○	X	X	○
**9 Sonstige Auffälligkeiten**					
9.1 Fehlende Krankheitseinsicht/schwere formale Denkstörung/R-L-Schwäche etc.					

Gesamteinschätzung des Risikos beim Führen eines Kraftfahrzeugs				
niedrig	mittel[1]	hoch[2]	sehr hoch[3]	nicht beurteilbar
○	○	○	○	○

Interpretationsrichtlinien: [1]maximal 2 niedrige oder 1 mittlerer Risikofaktor, [2]mehr als 2 niedrige oder mehr als 1 mittlerer Risikofaktor , [3]mindestens 1 hoher Risikofaktor

EvKB, Abteilung für Gerontopsychiatrie
